# Chinese medical teachers’ cultural attitudes influence palliative care education: a qualitative study

**DOI:** 10.1186/s12904-020-00707-w

**Published:** 2021-01-12

**Authors:** Antonia M. Willemsen, Piret Paal, Silja Zhang, Stephen Mason, Frank Elsner

**Affiliations:** 1 Faculty of Medicine, Department of Palliative Care, RWTH Aachen University, Pauwelsstraße 30, 52074 Aachen, Germany; 2 Institute of Nursing Science, Paracelsus Medical University, Salzburg, Austria; 3 Tongji Hospital, Huazhong University of Science and Technology, Wuhan, China; 4 Marie Curie Palliative Care Institute Liverpool, University of Liverpool, Liverpool, UK

**Keywords:** Palliative care, Education, Death, Culture, China, Global health

## Abstract

**Background:**

China holds one fifth of the world’s population and faces a rapidly aging society. In its ambition to reach a health care standard comparable to developed countries by 2030, the implementation of palliative care gains special importance. Until now, palliative care education in China is limited and disparate. This study aims to explore and determine factors that have impeded the development and implementation of palliative care education in China.

**Methods:**

We conducted semi-structured interviews with *n*=28 medical teachers from seven Chinese universities. Interviews were transcribed, and thematic analysis applied.

**Results:**

Three themes with two subthemes were constructed from data analysis. Theme 1 covers the still ambivalent perception of palliative care and palliative care education among participants. The second theme is about cultural attitudes around death and communication. The third theme reflects participants’ pragmatic general understanding of teaching. All themes incorporate obstacles to further implementation of palliative care and palliative care education in China.

**Conclusions:**

According to the study participants, palliative care implementation through palliative care education in China is hindered by cultural views of medical teachers, their perception of palliative care and palliative care education, and their understanding of teaching. The study demonstrates that current attitudes may work as an obstacle to the implementation of palliative care within the health care system. Approaches to changing medical teachers’ views on palliative care and palliative care education and their cultural attitudes towards death and dying are crucial to further promote the implementation of palliative care in China.

**Supplementary Information:**

The online version contains supplementary material available at 10.1186/s12904-020-00707-w.

## Background

Palliative care (PC) as defined by the World Health Organization (WHO) is an approach to improve the quality of life for patients with life-threatening illnesses and their families. It “prevents and relieves suffering through early identification, correct assessment and treatment of pain and other problems, whether physical, psychosocial and spiritual” [1].

Access to Palliative Care is a designated human right [2, 3]. The World Health Organization (WHO) promotes palliative care as an exclusive part of “the right to the enjoyment of the highest attainable standard of health and well-being” [4]. Still, in multiple countries in the world palliative care access remains low [5–7].

China holds one fifth of the world’s population and is the world’s largest developing country. The country faces demographic change, with an increasingly elderly population [5, 6, 8, 9]. Thus, the need for palliative care needs is growing commensurately and enabling nationwide access to palliative care is of special importance as China aims to reach health care standards comparable to developed countries by 2030 [10]. To do so will require a rapid realization of palliative care implementation.

Palliative care education is an essential step in implementing palliative care into a health care system [11] and providing palliative care to the people of a state [4]. The provision of palliative care education across the globe varies enormously [12] and palliative care education in China is limited and disparate [13, 14].

This study aims to explore and determine factors that affect the development and implementation of palliative care education in China. It is hypothesised that prevailing teachers’ beliefs, possible misconceptions and understanding of palliative care as a discipline, have a deleterious impact on education [15, 16].

## Methods

We conducted interviews with Chinese palliative care teachers and general medical teachers about their views on palliative care and palliative care education from Sep-Nov 2018. All interviews were conducted by the correspondent author. No repeat interviews were held. A COREQ (COnsolidated criteria for REporting Qualitative research) checklist can be found attached (Additional file 2).

### Interview schedule

A semi-structured interview schedule was developed for this study to explore Chinese palliative care teachers’ and general medical teachers’ views on palliative care and palliative care education. The initial guideline was developed in collaboration with palliative care experts and an expert on qualitative research methodology. Time for research in China was limited to 2 months, and therefore no pilot-testing could be conducted. Following the framework of grounded theory in terms of gaining ‘detailed descriptions’ and understanding ‘what lies beneath the surface’, but also to see ‘outside the pattern’ [17], hence, not to repeat the pattern, the interview schedule was adjusted according to interviewees responses multiple times. Questions covered self-presentation, questions about understanding of palliative care, palliative care practice, current palliative care education and its assessment, own palliative care education experiences, reasons for the lack of integration of palliative care within existing curricula, grief, communication about palliative care and expectations for the future. The first and the latest version of the interview schedule can be found attached as additional files (Additional file 1).

### Field notes

Field notes were taken to support the data analysis in terms of contextual interpretation of data and to assist in developing the themes and subthemes. Field notes covered details on how the contact came into being, interview setting, course of the interview, its atmosphere, feelings of the interviewer before, during and after the interviews, and reflections on the interviewer’s own performance.

### Interviewer

The corresponding author conducted all interviews. At the time of the field study, she was medical student in the 5th year. The interviewer had no experience in qualitative research and conducting interviews but was trained individually by co-authors F. Elsner and P. Paal.

In terms of expectations, the interviewer expected palliative care education structure in China to be low due to her previous work on a systematic review on the status of palliative care education in Mainland China [14]. Furthermore, difficulty to identify key people, possible language barrier as well as cultural barriers to discuss death and dying were expected [18]. No other expectations were present.

### Sample

The initial aim was to interview persons responsible for form and content of palliative care education at a university. However, such key persons barely existed due to little palliative care education at many universities in China. Often, palliative medicine was neither an explicit subject within medical curricula, nor represented by a department in hospitals. If practiced, palliative care was integrated within another specialty, for example oncology or traditional Chinese medicine. Therefore, it was often necessary to extend the sample and interview medical teachers working in the field of palliative care or general medical teachers.

The language barrier was an additionally complicating factor, resulting in difficulties in communication with hospitals and universities. Theoretical sampling through identification of key persons in palliative care education or palliative care teachers was thus difficult, and we used convenience and snowball sampling to find suitable participants.

Most possible participants were first approached by one of the co-author or members of the German Chinese Society of Medicine, who have a wide network of contacts to medical teachers in China. When possible participants showed general willingness for participation, direct contact to the interviewer was established either face-to-face, or through e-mail, telephone or a messenger application. A few participants were directly approached face-to-face by the interviewer.

According to the concept of information power, a concept proposed by Malterud et al. in order to in order to assess adequate sample size for qualitative studies [19], we estimated a rather high sample size needed to a representative sample. Thirty persons were asked for participation.

### Participant knowledge of the interviewer

Participants were informed about the goals of the study and the interviewer’s desire to publish. They also knew the interviewer’s occupation and the supporting institution, the German-Chinese Society of Medicine.

### Interview settings

To facilitate participation, participants identified suitable venues for the interview, with interviews held in offices within clinics in offices, meeting rooms, classrooms or hotel lobbies. One interview was held in a restaurant. On two occasions, participants had invited another person to the interview whom they considered palliative care expert or key person in education. Often, non-participants were present. Interviews duration range was 10 to 50 min.

### Language

Most interviews were conducted in English. Where participants spoke only Mandarin/Cantonese, translators or – occasionally – translating apps were used. Sometimes, participants spoke better German than English, or exclusively translators doing translations into German were available. In these cases, the interviews were held in German, the interviewer’s first language. Quotations from German language interviews used in this paper were translated into English after conducting the thematic analysis.

### Data analysis

As no previous studies stating theories on the subject of research existed [13, 14], we used thematic analysis as method for data analysis. This approach was particularly suitable, as it reflects implicit and explicit meanings within data in a descriptive, yet nuanced and complex way [20, 21]. Themes and subthemes were developed according to the principles outlined by Vaismoradi et al., who argue thematic analysis involves the search for and identification of common threads that extend across an entire interview or set of interviews [22]. The coding and analytical theme development process consisted of description and interpretation of data, both inductive (based on interview schedule) and deductive (based on field notes), emphasizing context and discourse.

Interviews were audio recorded and as the first step were fully transcribed, familiarizing the author with the data in the process. Initial ideas and thoughts were noted into memos. Transcripts and field notes were then reread and ‘meaning units’ or nodes essential to disambiguate the meaning within the (con) text marked with explicit or implicit ideas. These meaning units were subsequently coded, reducing and abstracting the original data into analytical themes. Reflection notes or memos were taken throughout analysis process. No software was used during the coding process.

In a second step, codes were assigned to groups along their similarities or possible generalizations. In that process, codes were compared, linked, and sometimes changed. Code groups were labelled with a word or phrase from the original data summarizing the main idea of each code group. Code groups were further linked and structured to build the first themes and subthemes. In a process of distancing from the data for some time and rereading the codes multiple times, themes and subthemes were rearranged and reconstructed multiple times to answer the research question in the best possible way.

The analysis took place without immediate peer checking. However, the correspondent author was frequently supervised by two co-authors involved in data collecting process. Findings were not sent back to participants to check or comment the findings.

## Results

From 30 persons asked for participation, two persons refused. In the end, 26 interviews with *n*=28 participants from seven Chinese universities were conducted. Participants’ characteristics can be found in Table 1.

**Table 1:** Characteristics of participants

Category	Number (***n***=28)
**Sex**	
Male	13 (46,4%)
Female	15 (53,6%)
**University**	
Huazhong University of Science and Technology, Wuhan	15 (53,6%)
Tongji University, Shanghai	3 (10,7%)
Peking University, Beijing	2 (7,1%)
University of Science and Technology, Hefei	5 (17,9%)
Southeast University, Nanjing	1 (3,6%)
Jinan University, Guangzhou	1 (3,6%)
South Medical University, Guangzhou	1 (3,6%)
**Function**	
Key person in palliative care education (responsible for form and content of palliative care education)	0
Key person in medical education (responsible for form and content of medical education)	7 (25%)
Medical Teacher working in the field of palliative care	10 (35,7%)
Medical Teacher	28 (100%)
**Positions**	
Hospital President	1 (3,6%)
Hospital Vice President	4 (14,3%)
Medical College Vice Director	1 (3,6%)
Chief of Teaching Office in Hospital with affiliated Medical College	1 (3,6%)
Department Director	3 (10,7%)
Department Vice Director	1 (3,6%)
Department Secretary of Teaching	1 (3,6%)
Medical College/University Professor	7 (25%)
Medical College/University Associate Professor	2 (7,1%)
Attending	
Oncology	7 (25%)
Respiratory and Critical Care Medicine	1 (3,6%)
Gynecology	1 (3,6%)
Gastroenterology	2 (7,1%)
Anaesthesia	2 (7,1%)
Traditional Chinese Medicine	1 (3,6%)
Thoracic Surgery	1 (3,6%)
Traumatology	1 (3,6%)
Orthopedic Surgery	1 (3,6%)
Traditional Chinese Medicine	2 (7,1%)
Radiation Oncology	3 (10,7%)
Pulmonology	1 (3,6%)
Nuclear Medicine	1 (3,6%)
Urology	1 (3,6%)
Nephrology	1 (3,6%)
Geriatrics	1 (3,6%)
Resident	
Geriatrics	1 (3,6%)
**Clinical Experience**	
> 15 years	14 (50%)
< 15 years	7 (25%)
No clinical experience	1 (3,6%)
Unknown	6 (21,4%)

Apart from the thematic analysis, the conduct of the study itself revealed some aspects on the structure of palliative care education. None of the hospitals visited for this study had a palliative care department. If palliative care was practiced, it was done so as part of another specialty such as oncology or traditional Chinese medicine. At none of the seven universities participants worked for, palliative care was taught as a distinct subject.

Three themes were derived from the data in the process of analysis. They show that the views of medical teachers build a barrier to the application and education of palliative care in China.

**Fig. 1:**
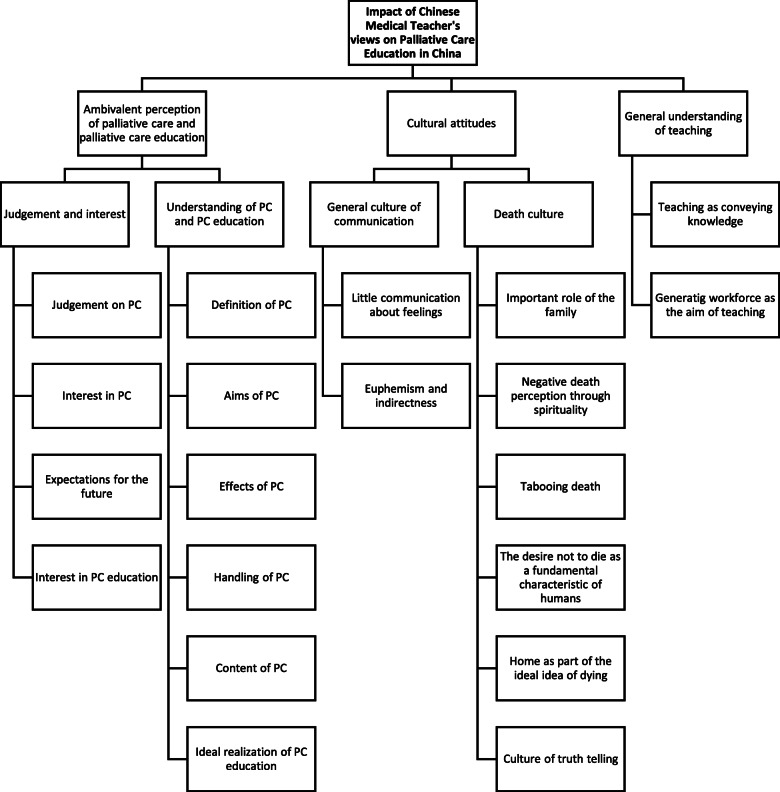
Coding tree

The first theme, “Ambivalent perception of palliative care and palliative care education”, includes the subthemes “Judgement on/interest in palliative care and palliative care education” and “Understanding of palliative care and palliative care education”. The second theme, “Cultural attitudes”, includes the subthemes “General culture of communication” and “Death culture”. The third theme, “General understanding of teaching” contains the two small subthemes “Teaching as conveying knowledge” and “Generating workforce as aim of teaching”. Subthemes may contain multiple aspects. An overview of themes, subthemes and individual aspects of subthemes can be found in Fig. 1 in form of a coding tree.

Overall, there was no remarkable difference between answers of participants with long clinical experience (> 15 years) or shorter clinical experience (< 15 years). In both groups, there was a great variety of answers and behaviour. No group showed a specific pattern or emphasized certain aspects.

### Theme 1: ambivalent perception of palliative care and palliative care education

The first theme shows that medical teachers in China have an ambivalent perception of palliative care and palliative care education. It includes two subthemes.

The first subtheme, “Judgements on/interest in palliative care and palliative care education” reflects the range of perceptions among medical or palliative care teachers. Some participants associated palliative care with enabling a peaceful way of dying, yet others with hopelessness.

*“Because palliative means no hope.” (T = Transcript 15)*

In the future, participants expected palliative care to play an increasingly important role in China’s health system, others were convinced that little changes are to be expected in the future due to a lack of interest among physicians. Some were contented with the status quo of palliative care education, some emphasized the need and desire for more palliative care practice and palliative care education in the future, and some showed no interest in the topic.

*“I think it’s, to be physician, if you want to be a physician, you must have this courses, because [ … ] You’re dealing with the dying patients. So, you should have this kind of knowledge. [ … ] You don’t need to be an expert, but you should know the knowledge about the palliative care.” (T23)*

Interestingly, it was noted during the interviews that several participants evidently felt uncomfortable discussing the subject of death and dying. Accordingly, this may indicate a lack of comfort or willingness to teach the subject.

*FN = Field Notes: „He seemed to be a bit uncomfortable when we actually pronounced the words ‘dying’ and ‘death’ but he managed it” (FN14)*

The second subtheme depicts participants’ understanding of palliative care and palliative care education. When asked about what palliative care is, most participants referred to symptom control. Others defined it through its application within the disease journey, mostly as part of oncology or as the last option of therapy.

*“Palliative medicine, we usually know that from the oncology department.” (T17)*

The ambivalent understanding of palliative care also became clear when talking about the aims of palliative care. Many participants mentioned quality of life and respect for the patient, but some also highlighted the goal to make the patient accept curative therapies.

*“[ … ] I would say, palliative care makes living better.“ (T11)*

*“Because chemotherapy, radiotherapy, or the surgery, they also bring vary kind of symptoms and adverse reactions for this kind of treatment. So, palliative treatment also help these kind of patients to have a good dealt with this kind of treatment. To accept the treatment more smoothly.” (T05)*

Those participants highlighting the importance of palliative care mentioned the benefits not only for the individual, but also for the families, the physicians, and the society.

“*On the other hand, it’s also a great burden for the family or for the city. If everyone thinks like that, operate, drugs, it’s also very expensive. Many families living on the countryside are financially very burdened, maybe. They have to sell houses. Misery again.” (T18)*

The interviews recorded various reactions to the subject of palliative care, for example grief and insecurity, highlighting the subject as taboo or, on the other side handling it with normality.

*“So, [the patient] didn’t want to change the therapy, and she want to come back in two months later and you know that the circle of therapy is three weeks to four weeks. So, after two months, then maybe the lesions grows up. We tried … I tried my best. (laughs) To give her suggestions and blablabla, and … she go back. (laughs)” (T03)),*

*FN: “He avoided the subject of palliative medicine [ … ]” (FN19)*

When talking about palliative care education, participants judged various addressees as ideal target group, such as nurses, young physicians, small children, oncologists or physicians in general. Views on the main emphasis in terms of content of palliative care education ranged from symptom control to communication.

*I = Interviewer: “If you would decide to teach palliative medicine [ … ], what would you consider especially important in teaching? What would you teach?” – P = Participant: “I think most important the symptoms. And then how you deal with them. That’s it.” (T01)*

More details and quotations on Theme 1 can be found in Table 2.

**Table 2:** Theme 1 - Ambivalent perception of palliative care and palliative care education

Subtheme	Aspect of Subtheme	Quotations
**Judgement and interest**	Judgement on PC → positive, unsure, negative	*- “I would like in peace go to die. […*] *Palliative medicine.” (T 17)* *- “So, I’m confused about the palliative care and aggressive care, because if you lose confidence for a patient, so just choose the palliative care for them, but confidence is the important thing for the patients. So, if the doctors lose confidence, and what the patient should do?” (T 15)* *- “Because palliative means no hope.” (T 15)*
	Interest in PC → existent, non-existent	*- Field notes (FN): “[…] she was interested in the topic and interested in the way palliative medicine was developing in China” (FN 24)* *- Field notes: “She just did not seem to care at all about palliative medicine” (FN 25)*
	Expectations for the future → change and need of change, little change	*- “So, I think it’s, the future, the palliative medicine will be, most people will pay attention to this field.” (T 20)* *“Now, it’s also the people are living longer, and have lots of the elders. And also, have lots of the cancer patients, or the Alzheimer’s disease, like this kind of disease. […] It’s we need the palliative medicine.” (T 20)* *“Most of the doctors, they don’t pay much attention to this topic.” (T 24)*
	Interest in PC education → desire for more, contented with status quo, lack of interest	*- “[…] the medical school, they should arrange some course. Like the palliative medicine.” (T 20)* *- “I think it’s, to be physician, if you want to be a physician, you must have this courses, because […] You’re dealing with the dying patients. So, you should have this kind of knowledge. […] You don’t need to be an expert, but you should know the knowledge about the palliative care.” (T 23)* *- Interviewer (I): „Do you think this [actual way of PC] is a good way of learning it [PC] […*]?*” – Participant (P): “Yes. I think this is a good way to teach them student.” (T 21)* *- I: “How do you think will the palliative care education develop in the future?” – P: “(moans)” (T 01)*
**Understanding of PC and PC education**	Definition of PC → contents: symptom control, death work, integral approach → usage: last option of therapy, part of oncology	*- „To control the symptoms for the patient. It is the main work of palliative treatment in the ward.” (T 05)* *- “Death education. […] Let each patient feel calm and calmer and accept death. He realizes that death is a process of life other than the end of life.” (T 10)* *- “[…] to try my best to make patient and their family members to be calm down to face the coming of the death.” (T 05)* *- “There are so many pains in patients, not only the body, but also the feelings and also some difficult situation in the society. […] And that’s also part of the palliative care.” (T 09)* *- “So, for the people, for the patient who have not method to cure their diseases, […] no medicine, no other method to cure them, this Pallimed medicine is to this patients.” (T 17)* *- “Palliative medicine, we usually know that from the oncology department.” (T 17)*
	Aims of PC → quality of life, respect for the patient and its will, acceptance of curative therapies	*- “[…] I would say, palliative care makes living better.* “*(T 11)* *- “Pay him this respect, respect. Give some respect. And make them feel comfortable.” (T 26)* *- “If the intention for this kind of patients is cure, I think palliative treatment is also an important part for this kind of patient treatment work. Because chemotherapy, radiotherapy, or the surgery, they also bring vary kind of symptoms and adverse reactions for this kind of treatment. So, palliative treatment also help these kind of patients to have a good dealt with this kind of treatment. To accept the treatment more smoothly.” (T 05)*
	Effects of PC → relief for physicians, families and hospitals	*- “At that time [when first dealing with PC patients], I feel helpless myself, because not only for them, but also for myself, I feel hopeless. But later, (chuckles) then I read about […] NCCN guidelines about palliative care, use more often […] Not treat their disease but make them feel better. I feel I learned a lot. Not only to treat the disease […] but I actually helped them.” (T 15)* *- “On the other hand, it’s also a great burden for the family or for the city. If everyone thinks like that, operate, drugs, it’s also very expensive. Many families living on the countryside are financially very burdened, maybe. They have to sell houses. Misery again.” (T 18)* *- “On the other hand, for the family it’s also an inner burden, then they can feel well: Oh, doing such thing is no bad thing.* “*(T 18)* *- “Seeing from the point of view of society in these times, it maybe is a relief for these hospitals. Because then, the people don’t go to the hospital that often, and they won’t stay that long. “(T 18)*
	Handling of PC → grief, insecurity, tabooing, normality	*- “When I know the disease can’t curable, so I’m very depressed. As a doctor I want to treat the patients. And help them get better and better.” (T 07)* *- “So, [the patient] didn’t want to change the therapy, and she want to come back in two months later and you know that the circle of therapy is three weeks to four weeks. So, after two months, then maybe the lesions grows up. We tried … I tried my best. (laughs) To give her suggestions and blablabla, and … she go back. (laughs)” (T 03)* *- FN: “He avoided the subject of palliative medicine […*]” *(FN 19)* *- “I’ve seen my patients here, and like I mentioned, the most patients here are in good, you know, conditions, but only a few guys is looking for, you know …*” *(T 01)* *- FN: “[The participant] Did not seem to feel inconvenient when I was asking about incurable patients” (FN 06)*
	Content of PC education → symptom control, communication	*- I: “If you would decide to teach palliative medicine […*], *what would you consider especially important in teaching? What would you teach?” – P: “I think most important the symptoms. And then how you deal with them. That’s it.” (T 01)* *- “I think this [introduction of courses in communication] is actually very important, because communication, it’s not only communication. The background, you actually have to learn a lot.” (T 04)*
	Ideal realization of PC education → various ideal addressees	*- “But we’re very sorry, the specialist courses are more in the field of nursing, not in clinical medicine. “(T 11)* *- “And I think, it’s also, first we’ll teaching the doctors.” (T 20)* *- „At first for medical student, we can start. And then we slowly have to, all young doctors have to learn this.” (T 04)* *- “So, I think, we should start the education in the very beginning, even in the small children. I just teach my boy, don’t scare about the death.” (T 23)* *- “Especially for some oncology or hematologist, they should have this knowledges about how to deal with patients.” (T 20)*

### Theme 2: cultural attitudes

The second theme identifies barriers to palliative care and palliative care education via the cultural attitudes of participants or their perception of cultural attitudes in society with the general culture of communication (subtheme 1) and death culture (subtheme 2) playing a major role.

Participants reported that communication with patients is often characterized by euphemism and indirectness and little conversation about feelings and emotions. These patterns were also present in the communication of participants themselves. This complicates communication about palliative care in both practice and education.

*I: “Can you describe me more about this feeling?” – P: “(laughs)” (T02)*

Death culture covers multiple aspects impeding palliative care application and education.

The role of family in society is huge, and equally so it is in dealing with death or making medical decisions at the end of life. Participants reported that families largely decide about medical aspects in disease at end of life. The social importance of family also pressures decision makers to favour curative approaches, as doing everything for the family is a high social ideal.

*“So, if your parents are ill, you cannot give them up. Because you will feel suffer yourself, you will think you are a bad person, and people around you will criticize you. Because you did the worst thing.” (T17)*

According to the participants, death has a very negative image within Chinese spirituality. Among both Buddhists and atheists, death is viewed as something tragic and very difficult to accept. Death and communication about it are socially stigmatized.

*“So, they [Chinese people] don’t think it’s good things to discuss death with others. Not polite. Very impolite.” (T24)*

The wish not to die is perceived as a basic characteristic of being human, and this results in an absolute and collective request for healing and curative treatment. Hospitals are viewed as healing places, where “bad results” and non-curative treatment options are very difficult to accept.

*“If she doesn’t want to live, then she will not go to the hospital.” (T03)*

Most patients who realize that they are going to die leave the hospitals to die at home or in community hospitals near their home. This contributes to palliative care being rarely conducted at university hospitals, providing students with little, if any experience or appropriate role models.

*I: „And what about other death related symptoms [ … ]. Are they present in education?“ – P: „Actually not [ … ]. Because most patients, they want to go home. They know, that’s incurable, they are going to die, they won’t die here at the hospital.“ (T04)*

The culture of truth telling or discussing prognosis is often referred to as an obstacle to palliative care. Participants had varying views on the culture of truth telling. Some supported and accepted it, whilst others judged it negatively. Still, most participants reported acting according to the family’s wishes, who generally ask physicians not to inform terminally ill patients about diagnosis and prognosis.

*„[ … ] the family always tell the doctor, don’t tell (chuckles) the truth to the patient. So, I respect the family [ … ]” (T19)*

Information disclosure though is seen as a precondition for practicing palliative care, and the culture practices in relation to truth telling therefore work as a barrier for palliative care practice in China.

*“But whether or not, whether to receive the palliative treatment or not is decided by comprehensive reasons, like economic status. [ … ] And psychology status. How his ability to tolerate or accept the truth.” (T16)*

More details and quotations on Theme 2 can be found in Table 3.

**Table 3:** Theme 2 - Cultural attitudes

Subtheme	Aspect of subtheme	Quotations
**General culture of communication**	Little communication about feelings	*- “So, I think Chinese patient is very … they are very ashamed to express their feelings. And some Chinese patient, they don’t want to take much trouble to the family members, so I think many patient concealed their feelings.” (T 05)* *- I: “Can you describe me more about this feeling?” – P: “(laughs)” (T 02)*
	Euphemism and indirectness	*- “Because Chinese … (types on smartphone for translation) […] Euphemistic! […] In general, they are euphemistic and indirect. […] When they face the incurable disease […]” (T 22)* *- “I will give him [the patient] some choice to choose, for example, to treat very positively or, it’s not abandon, but it’s softly treat.” (T 02)*
**Death culture**	Important role of the family → in handling disease → as decision maker → high social value of family as moral guide	*- “For example, they [relatives] also are in Chinese clinic. All family members are there, yes. […] But in Germany … […] Totally different. The patients stay in bed alone, maybe once the family visits in the hospital.” (T 04)* *- “But in my experience, mostly it’s the family makes decision.” (T 07)* *- “So, if your parents are ill, you cannot give them up. Because you will feel suffer yourself, you will think you are a bad person, and people around you will criticize you. Because you did the worst thing.” (T 17)*
	Negative death perception through spirituality	*- “Death, to death, is horrible to us, that is eastern people. But in the west, they can more peaceful to this situation, to see dying, to see God.” (T 17)* *- “So, in China, many, many patients is afraid of death. It’s not like the west. And many of this patient, they don’t have religion, they don’t believe. […] So, we can’t talk too clear direct to the patients: You have no time, six months or so … like so.” (T 08)* *- “[…] most of them have this religion in Buddhism. But yours are Christian […] You know, in Christian, maybe from your child, you’re educated that death is part of your life. […] But for Chinese people, most of them think death is very difficult to accept.” (T 24)*
	Tabooing death	*- “So, they [Chinese people] don’t think it’s good things to discuss death with others. Not polite. Very impolite.” (T 24)*
	The desire not to die as a fundamental characteristic of humans → consequence of absolute cultural request for healing and curative treatment → hospitals as answer to this request	*- “Because people don’t want to die, right?” (T 02)* *- “And following our tradition, the relatives, especially the daughters and sons, who simply … they think that keeping the patient alive, that’s important. “(T 04)* *- “[…] at the beginning, as young doctors, they only pay attention to the technology, and the main thing is I healed this patient, this disease.” (T 04)* *- “If she doesn’t want to live, then she will not go to the hospital.” (T 03)* *- “A lot of people in this country think, they just come to a hospital, they have to get the good result.” (T 24)*
	Home as part of the ideal idea of dying	*- I: „And what about other death related symptoms […*]. *Are they present in education? “– P: „Actually not […]. Because most patients, they want to go home. They know, that’s incurable, they are going to die, they won’t die here at the hospital. “(T 04)*
	Culture of truth telling → Information disclosure as precondition to PC → ambivalent judgement	*- “But whether or not, whether to receive the palliative treatment or not is decided by comprehensive reasons, like economic status. […] And psychology status. How his ability to tolerate or accept the truth.” (T 16)* *- I: „Do you talk with patients about death? Or with families about death? And dying?” – P: “But for the patient, we usually don’t directly talking this. […] Most, I will want give the patients hopes.” (T 20)* *- “In my opinion, I think the patient should know the truth of their disease.” (T 22)* *- “So, in China, we don’t want to talk, in your life, we don’t to talk about the death […] But it’s a real life. We must face it.” (T 23)*

### Theme 3: general understanding of teaching

The third theme describes participants’ general understanding of teaching. Teaching was mostly understood as the conveyance of knowledge and the primary aim was to generate workforces to meet the needs of the state.

“*That’s our main emphasis, to enable the students to learn, to master this knowledge.” (T04)*

*“First, it has to meet the need of the state. We need more physicians.“ (T18)*

## Discussion

### Summary

Results from our study suggest that medical teachers’ views impede the application and education of palliative care.

The prevailing ambivalence in the perception of palliative care among the participants shows different levels of openness to both application and education. This includes ambivalent judgements on palliative care and interest in the topic, ambivalent views on aims and effects, as well as ambivalent definitions and reactions.

The culture-bound views of both medical teachers and society impede palliative care and palliative care education. The general culture of “death as a taboo” complicates communication about end of life care, where exchange is centred on the high social value of the family and a negative cultural and spiritual understanding of death. As patients prefer to die at home or near home and seek help mostly in community hospitals, the number of palliative care patients in university hospitals is low. Restrictions to discuss the prognosis further than “the patient is at the end of life” impede communication about the provision of palliative care as an option at the end of life.

The general understanding of the purpose and function of teaching is very pragmatic. Conveying knowledge is the centre of education and its primary aim is the generation of a competent workforce. This may be a barrier for holistic and reflective approaches such as palliative care to establish a role within medical education.

### The relation of culture and palliative care

The general effect of culture as a barrier to palliative care education has received little direct attention until now. Yet, associations between culture and palliative care have been subject to research for years. For example, culture and culture-bound belief systems play a major role in the provision of high-quality palliative care [23–25]. Appreciating cultural variations in fundamental human concepts such as meaning of life, suffering, quality of life and communication patterns is essential to provide holistic end-of-life care [25]. Our study demonstrates the impact of culture on the quality of palliative care itself, as well as on the implementation of palliative care into a health care system through palliative care education. Our data and analysis suggest that palliative care education in China is impeded by the social value of family and the need to maintain family harmony, by the negative cultural and spiritual perception of death with its consequence of social stigma, and by general practices of communication.

### The culture of truth telling

As our participants report, a culture of withholding information on diagnosis and prognosis from terminally ill patients in preference for the family, is largely practiced in China. Information is first communicated to the family, who will mostly ask the physician to withhold this from the patient for fear of doing harm: a practice widely described in literature [26, 27]. In our interviews, participants explained their rationale for this approach through the ethical principles of beneficence and maleficence [28].

In recent years, the Chinese culture around truth telling within end of life care has been widely discussed. Research shows that patients prefer information disclosure more often than their families [29] or physicians [30]. Chinese patients without clear awareness of their diagnosis and prognosis experience more anxiety and difficulty in communication with their families [31]. These studies indicate that information disclosure might not harm Chinese patients but be in their interest. The fact that our participants named Chinese truth telling culture as one barrier to palliative care application and education undermines this thesis. This indicates that nurses and physicians need palliative care education to be able to support and educate families. Where the benefits and harms are clear, the withholding of information on diagnosis and prognosis seems more than questionable.

### General understanding of teaching

Teachers’ understanding of their role has an impact on the quality of education [16]. In our study, most participants understand teaching as the conveyance of knowledge. However, when it comes to palliative care, teaching goals go beyond that. The European Association for Palliative Care (EAPC) names the understanding of holistic treatment as one goal of palliative care education. Another goal is to show that reflection upon own attitudes towards disease, death, dying and mourning is essential to provide quality end-of-life care [32]. Students value the experience and clinical relevance of understanding patients through a holistic lens, a process that is championed within palliative care education [33, 34]. Reflection in education leads to more positive learning experiences [35] and is essential to be able to transfer the knowledge to practical, unknown situations [36]. Students evaluate reflection in palliative care education as positive, enabling them to learn about their own limitations [33], their personal development and to deepen humanistic aspects in their practice [34]. These elements of reflective, holistic, person centred care are not yet mainstream within Chinese medical education.

### Information power

Malterud and colleagues have argued that the prevailing concept for sample size in qualitative studies is “saturation.” They also have pointed out that this term is inconsistently applied and proposed the concept “information power” in order to guide adequate sample size for qualitative studies. Accordingly, information power indicates that the more information the sample holds, relevant for the actual study, the lower amount of participants is needed [19]. Following this statement, we estimated a rather high number of interviews to be needed to have valid results after data analysis. With *n*=28 participants, we believe that enough data were collected to have valid results. Still, we cannot rule out that more interviews could have revealed additional information.

### Strengths and limitations

This study is the first to examine medical teachers’ views and perceptions on palliative care and palliative care education. It enables a better understanding of the role of palliative care in the Chinese medical educative system and possible reasons for the slow implementation of palliative care education. This understanding plays an important part inone day being able to overcome the existing obstacles for the implementation of nationwide palliative care education and through this for achieving nationwide access to high quality palliative care.

Interviews were held in different regions in China at multiple universities, and participants held various positions. Through this, our data gives a very rich picture on participants’ views and perceptions on palliative care and palliative care education. This is enforced through the method of thematic analysis, which enables a complex and detailed reflection of interview contents.

At the same time, this study is subject to various limitations.

Findings are at risk of language and interpretive bias. Interviews were not held in participants’ mother tongue. Further, the use of translators may have resulted in small changes of meaning and nuance, particularly as translators were mostly non-professional and not familiar with core characteristics of qualitative research.

Another weakness of the study is the sample generation. Due to the limited structure of palliative care education in China, it was difficult to identify key persons in palliative care education or general medical education. We often relied on snowball or convenience sampling to conduct interviews. Therefore, sample specificity is limited.

Interviews were possibly influenced by noisy settings, disruptions and on occasion the presence of an uninvolved third person, possibly exerting social pressure. Indeed, the interview itself might have exerted a form of social pressure, as participants might have experienced discrepancies in palliative care knowledge between them and the interviewer. Some participants gave the impression of feeling ashamed about their little palliative care knowledge; some appeared to feel uncomfortable to discuss end-of-life subjects due to social stigma. Additionally, social desirability in form of participants aiming to please the interviewer might well have affected participants’ statements and answers, despite the interviewer’s efforts to show an open and unjudging mind frame.

Additionally, the interviewer was rather new to the interviewer’s role. Therefore, information power of some interviews is limited, and sympathy, antipathy or personal views may have influenced the course of interviews. As thematic analysis was conducted by only one author, subjectivity may have influenced results.

It has been suggested that thematic analysis, as a flexible and useful research tool, provides a rich and detailed, yet complex, account of the data. Thematic analysis is “a reiterative process without finite interpretation”, however, it is a key methodology when it comes to generating hypotheses and theories [22]. As only one author conducted thematic analysis, subjectivity may have influenced results. However, the analysis was conducted over several time-points, which is believed to add objectivity to the final themes and subthemes presented and discussed in this paper.

## Conclusions

Currently, palliative care implementation through palliative care education in China is hindered by prevailing cultural views. The prevailing culture promotes ambivalent perceptions of palliative care among medical teachers and the wider society. A pragmatic general understanding of teaching works as further barrier for palliative care education, which is elsewhere characterized by holistic approaches, communication, and reflection. However, developing research is challenging the cultural orthodoxy and highlighting the value of rethinking end of life culture, reducing harm not through withholding diagnosis and prognosis, but by enabling high quality end of life care through a more open communication.

Approaches, such as train the trainer sessions, to change medical teachers’ views on palliative care and palliative care education and their cultural attitudes towards death and dying are crucial to further promote the implementation of palliative care in China.

## Supplementary Information

**Additional file 1.** “Interview Schedule – First and Final Version”, format: .pdf, first and final version of the semi-structured interview schedule used in the interviews, final version as product of multiple adjustments of the first version.

**Additional file 2.** “COREQ-Checklist”, format: .pdf, a list of COREQ-guidelines for reporting qualitative studies with all requested information and references where to find them in the manuscript.

## Data Availability

The datasets generated and/or analysed during the current study are not publicly available due the protection of participants’ identities but are available from the corresponding author on reasonable request.

## Abbreviations

PC: Palliative care
I: Interviewer
P: Participant
T: Transcript
FN: Field Notes
