# Supplementary material for: Chinese medical teachers’ cultural attitudes influence palliative care education: a qualitative study
Source: BMC Palliat Care. 2021 Jan 12;20:14. doi: 10.1186/s12904-020-00707-w (PMC7805147; doi:10.1186/s12904-020-00707-w)
Supplement: Supplementary file 1 — Additional file 1. “Interview Schedule – First and Final Version”, format: .pdf, first and final version of the semi-structured interview schedule used in the interviews, final version as product of multiple adjustments of the first version. [file 12904_2020_707_MOESM1_ESM.pdf]

<u>Additional file 1</u>

Interview Schedule – First Version

Self-presentation

1. What position do you have at your university?

2. What is your role in general medical education and in PC education?

Depiction of personal views

3. Personal understanding of PC: What does PC mean to you?

    - *In how far differs PC treatment from treatment in other departments?*
    - *Personal experiences? When was your first contact, how did it develop over time?*
    - *Did you miss something?*
    - *Did you receive a form of education in PC?*
    - *What do you think of PC?*

4. Personal understanding of teaching: How do you see yourself as a teacher?

    - *How do you teach students?*
    - *What do you consider important aspects when teaching students?*

Status of PC education at your university

5. Please describe PC education at your university

    - *What do you think of the PC education at your university? (How do you assess it?)*

If there is PC education, continue with 6a. If not, continue with 6b.

6.

a) You just told me what aspects you consider important when teaching students. Are there any aspects that are especially important when teaching palliative care?

    - *Main competencies a student should learn?*

- *How do you teach in order to achieve that?*

b) If there is no PC education at your university: Imagine you wanted to introduce PC education at your university. What would you have to do?

- *What would be the necessary steps?*
- *What would be needed?*
- *What would be possible obstacles?*

7. How will the future of PC education at your university be like?

- *What are your hopes for the future?*
- *Is there a need for change?*
- *What are needs for further development? (only if PC education already existing)*
- *What are obstacles to further development? (only if PC education already existing)*

Status of PC education in China

8. How is the situation of PC education in China in general?

- *What do you think of it? (How do you assess it?)*

9. What reasons could you imagine why PC education is not further implemented in China?

10. Does the government promote the introduction of Palliative care and PC education?

- *If so, how?*

11. How do you imagine the future of PC education in China in general?

- *In how far differ the needs and obstacles for further development from the ones at your university?*
- *What are your hopes?*

12. Is there anything you would like to add?

Interview Schedule – Final Version

Self-presentation

13. What position do you have at your university?
    - Since when do you work/teach?
    - What do you teach?
    - How do you teach? Give examples.
    - What do you consider especially important when teaching in general?

Depiction of personal views

14. What does the term of Palliative Medicine mean to you?
    - Do you have contact to such patients?
    - How is that like? Please describe the experience.
    - Do you remember your first contact? How was it like? Please describe.
    - Do you teach it?
    - What do you consider important when teaching it?
    - How would you assess this way of teaching it? What should be different? Why?

15. How did you yourself learn how to deal with such patients?

16. What do you think are the reasons why this topic, dealing with such patients, is not taught more systematically?

17. What do you think are the reasons why this topic is not already taught to students at university?

18. When does … get taught here? How?
    - Pain management?
    - Ethics?
    - Communication?
    - Death related symptoms (other than pain)?
    - Do you talk about grief during your medical education?

19. What are the options for patients where curative treatment is not possible anymore? Who decides?
20. How do you talk to patients about palliative treatment?
21. How will the future be for Palliative Treatment and PC education at your university?
    - What are your hopes?
22. Is there anything you would like to add?
