# Supplementary material for: Chinese medical teachers’ cultural attitudes influence palliative care education: a qualitative study
Source: BMC Palliat Care. 2021 Jan 12;20:14. doi: 10.1186/s12904-020-00707-w (PMC7805147; doi:10.1186/s12904-020-00707-w)
Supplement: Supplementary file 2 — Additional file 2. “COREQ-Checklist”, format: .pdf, a list of COREQ-guidelines for reporting qualitative studies with all requested information and references where to find them in the manuscript. [file 12904_2020_707_MOESM2_ESM.pdf]

<u>**Additional file 2**</u>

COREQ-Checklist for the manuscript "Chinese Medical Teachers' Cultural Attitudes influence Palliative Care Education: A Qualitative Study"

No.	Item	Requested Information	Reference in Manuscript
**Domain 1: Research team and reflexivity**			
Personal characteristics			
1.	Interviewer/facilitator	Antonia Willemsen, correspondent author	Methods: Interviewer (p. 5)
2.	Credentials	None	Title page (p. 1)
3.	Occupation	Medical student	Methods: Interviewer (p. 5)
4.	Gender	Female	Methods: Interviewer (p. 5)
5.	Experience and training	The interviewer was trained individually by co-authors F. Elsner and P. Piret	Methods: Interviewer (p. 5)
Relationship with participants			
6.	Relationship established	No special relationship. Contact about time, place and issue before the interviews was mostly carried out via E-Mail or messenger application.	Methods: Sample, paragraph 3 (p. 6)
7.	Participant knowledge of the interviewer	Participants knew the goals of the study and the interviewer's desire to publish. They also knew the interviewer's occupation and her supporting institution, the German-Chinese Society of Medicine.	Methods: Participants' knowledge of the interviewer (p. 6)
8.	Interviewer characteristics	The interviewer expected PC education structure in China to be low due to her previous work on a systematic review. She also expected difficulties in terms of sampling, language barrier and cultural barriers. No other expectations, to her best knowledge.	Methods: Interviewer (p. 5)
**Domain 2: study design**			
Theoretical framework			

9.	Methodological orientation and Theory	Thematic analysis	Methods: Data analysis, paragraph 1 (p. 7)
Participant selection			
10.	Sampling	Convenience and snowball sampling	Methods: Sample, paragraph 2 (p. 5)
11.	Method of approach	First approach: mostly by one of the co-authors, or members of the German Chinese Society of Medicine, then contact between interviewer and possible participant via e-mail, telephone or a messenger application. Sometimes direct first face-to-face contact between interviewer and participant.	Methods: Sample, paragraph 2 (p. 5)
12.	Sample size	n=28	Results, paragraph 1 (p. 8)
13.	Non-participation	n=2	Results, paragraph 1 (p. 8)
Setting			
14.	Setting of data collection	In clinics: offices, meeting rooms, classrooms<br>In hotels: lobbies<br>In one restaurant	Methods: Interview settings (p. 6)
15.	Presence of non-participants	Often	Methods: Interview settings (p. 6)
16.	Description of sample	Characteristics of participants	Table 1 (p. 9)
Data collection			
17.	Interview guide	Interview guideline attached in separate file. No pilot testing.	Attached File 1: First and final version of interview schedule, Methods: Interview schedule (p. 4)
18.	Repeat interviews	None	Methods: Paragraph 1 (p. 4)
19.	Audio/visual recording	Audio recording	Methods: Data analysis, paragraph 2 (p. 7)
20.	Field Notes	Done	Methods: Field Notes (p. 4)
21.	Duration	10-50 Minutes	Methods: Interview settings (p. 6)
22.	Data saturation	According to the concept of information power: enough interviews for valid results	Discussion: Information power (p. 18)

23.	Transcripts returned	None	Methods: Data analysis, paragraph 4 (p. 8)
**Domain 3: Analysis and findings**			
Data analysis			
24.	Number of data coders	1	Methods: Data analysis, paragraph 4 (p. 8)
25.	Description of the coding tree	Figure 1	Figure 1
26.	Derivation of themes	Derived from data	Results, paragraph 3, (p. 8)
27.	Software	No software used	Methods: Data analysis, paragraph 2 (p. 7)
28.	Participant checking	None	Methods: Data analysis, paragraph 4 (p. 8)
Reporting			
29.	Quotations presented	Yes	Results: Theme 1, Theme 2, Theme 3 (p. 10-15)
30.	Data and findings consistent	Yes	Results: Theme 1, Theme 2, Theme 3 (p. 10-15)
31.	Clarity of major themes	Yes	Results: Theme 1, Theme 2, Theme 3 (p. 10-15); Table 2 and Table 3 (p. 27-30)
32.	Clarity of minor themes	Diversity of participants' attitudes included in the themes	Results, paragraph 4 (p. 8)
